# Synthesis of Five Known Brassinosteroid Analogs from Hyodeoxycholic Acid and Their Activities as Plant-Growth Regulators

**DOI:** 10.3390/ijms18030516

**Published:** 2017-03-08

**Authors:** María Isabel Duran, Cesar González, Alison Acosta, Andrés F. Olea, Katy Díaz, Luis Espinoza

**Affiliations:** 1 Departamento de Química, Universidad Técnica Federico Santa María, Avenida España 1680, Valparaíso 2340000, Chile; maria.duranp@alumnos.usm.cl (M.I.D.); cesar.gonzalez@usm.cl (C.G.); 2 Escuela de Ingeniería en Biotecnología, Facultad de Ciencias Biológicas, Universidad Andrés Bello, Quillota 980, Viña del Mar 2520000, Chile; al.acosta@uandresbello.edu; 3 Instituto de Ciencias Químicas Aplicadas, Facultad de Ingeniería, Universidad Autónoma de Chile, Santiago 8910339, Chile; andres.olea@uautonoma.cl

**Keywords:** brassinosteroid analogs, synthesis, plant-growth regulators, lamina inclination test

## Abstract

Brassinosteroids (BRs) are plant hormones that promote growth in different plant organs and tissues. The structural requirements that these compounds should possess to exhibit this biological activity have been studied. In this work, a series of known BR analogs **5**–**15**, were synthesized starting from hyodeoxycholic acid **4**, and maintaining the alkyl side chain as cholic acid or its methyl ester. The growth-promoting effects of brassinolide (**1**) and synthesized analogs were evaluated by using the rice lamina inclination assay at concentrations ranging from 1 × 10^−8^–1 × 10^−6^ M. Our results indicate that in this concentration range the induced bending angle of rice seedlings increases with increasing concentration of BRs. Analysis of the activities, determined at the lowest tested concentration, in terms of BR structures shows that the 2α,3α-dihydroxy-7-oxa-6-ketone moiety existing in brassinolide is required for the plant growing activity of these compounds, as it has been proposed by some structure-activity relationship studies. The effect of compound **8** on cell elongation was assessed by microscopy analysis, and the results indicate that the growth-promoting effect of analog **8** is mainly due to cell elongation of the adaxial sides, instead of an increase on cell number.

## 1. Introduction

Brassinosteroids (BRs) are a naturally occurring polyhydroxysteroidal plant hormone group that regulates plant growth and development by producing an array of physiological changes [1]. Natural BRs occur at low concentrations throughout the plant kingdom, with a range of 1–100 µg·kg^−1^ fresh weight, while shoots and leaves usually contain even lower amounts, i.e., 0.01–0.1 µg·kg^−1^ fresh weight [2]. They have been detected in all plant organs (pollen, anthers, seeds, leaves, stems, roots, flowers, and grains) and also in the insect and crown galls. Further work has demonstrated that BRs elicit a broad spectrum of physiological and morphological responses in plants, including stem elongation, leaf bending and epinasty, induction of ethylene biosynthesis and proton pump activation, synthesis of nucleic acid and proteins, regulation of carbohydrate assimilation and allocation, and activation of photosynthesis [1]. The leaf bending caused by extracts from *Distylium racemosum* in the rice lamina inclination assay is one of the earliest reports of brassinolide (**1**) activity in plants [3]. In this assay, **1** caused dose-dependent swelling of the adaxial cells in the joint between the leaf blade and sheath of etiolated rice seedlings. The angle of the leaf bending caused by the swelling is used as a sensitive bioassay during brassinolide isolation and quantification procedures [4]. An early report on growth-promoting activity of “brassin”, from which **1** was isolated, was based on brassinolide-mediated stimulation of bean elongation at the 2nd and 3rd internodes, with higher concentrations causing the splitting of the stem [5,6]. Brassinolide and synthetic analogs were subsequently used in various bioassays, which indicated that in addition to leaf bending, cell elongation, and cell division, **1** also affects source/sink relationships [7], proton pumping and membrane polarization [8,9], photosynthesis [10] and stress responses including thermotolerance [11,12] and senescence [13]. The role of BRs in improving the productivity of various crop plants such as vegetables, fruits, and oil seed plants are well documented [14,15,16,17].

**Figure 1:**
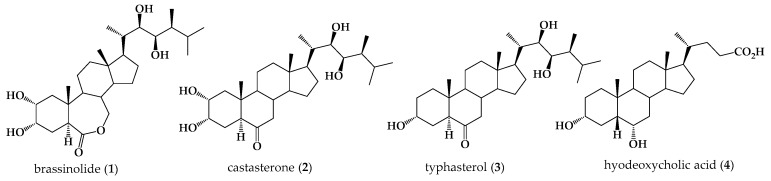
Brassinolide (**1**), castasterone (**2**), typhasterol (**3**) and hyodeoxycholic acid (**4**).

All natural bioactive BRs possess a vicinal 22*R*, 23*R* diol structural functionality (for example brassinolide (**1**), castasterone (**2**) and typhasterol (**3**), Figure 1), which seems to be essential for high biological activity. In this sense, several studies have been focused on determining the structural requirements that these compounds should possess to elicit strong biological activity [18,19,20]. It has been concluded that the activity of BRs depends on the oxygen atoms spatial situation, and a new way to relate the structure of BRs with their activity has been proposed [21,22]. Namely, the activity is attributed to the different conformations that BR molecules can adopt, one of them being the active conformation. Therefore the structural requirements are not associated to the presence or absence of a specific functional group in the molecule [18,19], but to the spatial distribution of all the functionalities present in it [22]. These spatial orientations can be indicated as distances or angles between the oxygen atoms present in a brassinosteroid. However, in recent decades, efforts have been focused on the synthesis of new BR analogs, keeping common patterns of organic functions in the A/B rings and *cis-trans* fusion between them, as occurs in some natural BRs, but with moderate or dramatic structural changes (shorter side chains, different oxygenated functions, spirostanic, aromatic and cyclic substituents, methyl esters, carboxylic acids) [21,23,24,25]. Surprisingly, some of these analogs have presented very important biological activity.

**Figure 2:**
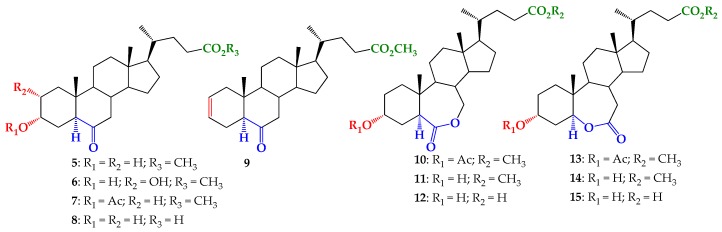
Brassinosteroid analogs obtained from hyodeoxycholic acid (**4**).

Therefore, in this work we report the synthesis of a series of known brassinosteroid analogs, (**6**, **7**, **9**, **10**, **12**, **13** and **15**), starting from hyodeoxycholic acid (**4**) and following a procedure recently described for the synthesis of analogs **5**, **8**, **11**, and **14** (see Figure 1 and Figure 2) [26]. In all these compounds **5**–**15** the native side chain is kept as cholic acid or its methyl ester. In addition, the effects of analogs **5**, **6**, **8**, **12** and **15** on elongation in the rice lamina inclination assay were evaluated at concentrations ranging from 1 × 10^−8^–1 × 10^−6^ M. The results indicate that analogs **12** and **15** are the more active compounds in the applied test at the same concentration (1 × 10^−8^ M). Additionally, in this work we inform the full NMR spectroscopic characterization for these derivatives, which was previously reported only in partial form.

## 2. Results and Discussion

### 2.1. Chemical Synthesis

Hyodeoxycholic acid (**4**) has been used for the synthesis of brassinolide and a number of analogs because it contains the organic functions, which can be chemically modified, at suitable positions in order to fulfill the structural requirements for their activity [27].

**Scheme 1:**
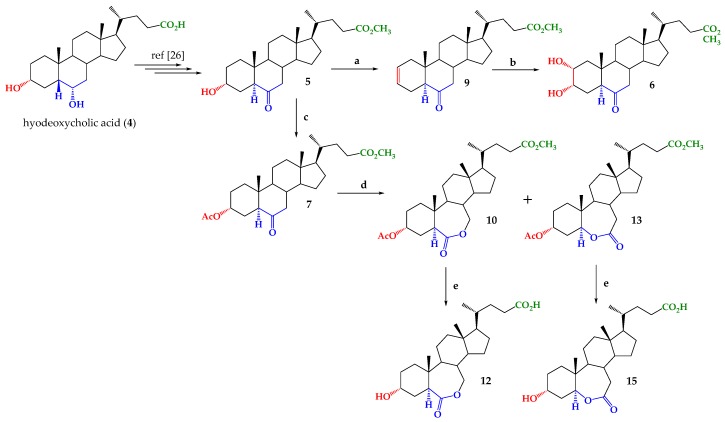
Synthesis of hyodeoxycholic acid derivatives **5**, **7**, **9** and brassinosteroid analogs **6**, **10**, **12**, **13** and **15**. *Reagents and conditions*: (**a**) Tf_2_O/DMAO/CH_2_Cl_2_, 0 °C, 24 h.; (**b**) OsO_4_ 4%/NMO, acetone, r.t., 72 h.; (**c**) Ac_2_O/DMAP, CH_2_Cl_2_/py, r.t., 30 min.; (**d**) (CF_3_SO_2_)_2_/H_2_O_2_/CHCl_3_, 0 °C, 24 h.; (**e**) K_2_CO_3_ 15% (aqueous solution)/CH_3_OH, reflux, 1 h. Derivative 5 has been previously reported [26].

Compound **5**, with 5α-cholestan-6-one skeleton (Scheme 1), has been synthesized by different methods [26,28,29,30,31,32], but we have obtained it from hyodeoxycholic acid (**1**) following a procedure previously described by us [26]. Compound **9** is obtained from **5** through a dehydration reaction in which the alcohol group at C-3 position of compound **5** is transformed into the double bond at C-2 position of compound **9**. A large number of reports have shown that the indirect or direct conversion of 3α or 3β-hydroxy steroid in Δ^2^ alkenes can take place by following at least three different strategies. The indirect path transforms the alcohol into a good leaving group such as tosylate, which in a subsequent treatment with Li_2_CO_3_/LiBr/DMF in reflux system [25,28,33,34,35] or treatment of mesylate derivatives under the same conditions produces the Δ^2^ alkenes [26,36,37,38,39]. On the other hand, an efficient alternative is the direct dehydration of 3α or 3β-hydroxy steroid that is carried out by adsorbing the substrate on silica gel in a reaction catalyzed by copper (II) sulfate [32,40,41,42]. In this work, the conversion of compound **5** to **9** was performed by using a mild *one-pot* method with Tf_2_O/DMAP in CH_2_Cl_2_ at 0 °C, and the alkene **9** was obtained with 83% yield (Scheme 1) [43]. The spectroscopic data (IR, ^1^H and ^13^C-NMR) and physical properties of compound **9** were consistent with those reported [26,28,32]. Compounds **6** and **7** were synthesized from **9** and **5** respectively, according to the methodology previously described by us [26].

**Figure 3:**
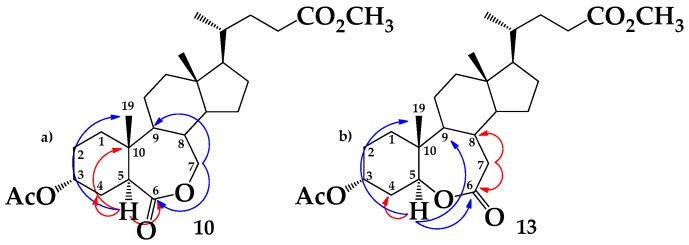
Major observed ^2^*J*_HC_ (**red arrows**) and ^3^*J*_HC_ (**blue arrows**) correlations of hydrogens H-5α and H-7 for compound **10** (7-oxalactone derivative (**a**)) and compound **13** (6-oxolactone derivative (**b**)).

The synthesis of lactones **11** and **14** from 3α-hydroxy-6-oxo-5α-cholanate **5** has been previously reported [26] (Figure 2). In this case, the Baeyer-Villiger oxidation of **5** with *m*-CPBA/CH_2_Cl_2_ gave a mixture of known 7-oxalactone **11** and 6-oxalactone **14** with 10.8% and 14.9% yields, respectively [26]. Other authors reported the synthesis of these compounds with 26% and 44% yields respectively [29]. However, it is known that oxidation of 5α-6-keto-steroid would result in the stereo and regioselective formation of the 7-oxalactone when electron-withdrawing substituents are present in the C-3 positions. Also, the use of CF_3_CO_3_H as an oxidant agent has a marked effect upon the 6-oxa:7-oxa ratio, and can lead to the preferential formation of the desired 7-oxa isomer [44,45,46]. For these reasons we carry out the Baeyer-Villiger oxidation of compound **7** with CF_3_CO_3_H/CHCl_3_ system. In this case the known 7-oxalactone **10** and 6-oxalactone **13** (Scheme 1) were obtained with 44% and 8% yields, respectively. Even though the synthesis of compounds **10** and **13** was previously reported, no NMR and IR spectroscopic data of **t**hese compounds were included [15]. Thus, the full structural assignment of compound **10** was mainly assigned by ^1^H, ^13^C, 2D Heteronuclear Single Quantum Coherence (HSQC) and 2D Heteronuclear Multiple Bond Correlation (HMBC) NMR spectroscopy. In the ^1^H-NMR spectrum of compound **10** a signal was observed at *δ*_H_ = 4.10–4.07 ppm (m, 2H), which was assigned to the two hydrogens H-7 and correlated by 2D ^1^H-^13^C HSQC with the signal at *δ*_C_ = 70.47 ppm (CH_2_-7 from ^13^C and DEPT-135 spectra, Table 1). Additionally, from 2D ^1^H–^13^C HMBC spectrum important heteronuclear correlations were observed: (i) H-7 shows ^3^*J*_HC_ correlations with the signal at *δ*_C_ = 176.11 that was assigned to the carbon C-6 (C=O of lactone function, Table 1), and the signal at *δ*_C_ = 58.43 ppm, assigned to the carbon C-9; (ii) H-5α at *δ*_H_ = 3.02 ppm (1H, dd, *J* = 12.1 and 4.0 Hz) showed ^3^*J*_HC_ correlation with signals at *δ*_C_ = 14.58, and *δ*_C_ = 58.43 ppm (Table 1), which were assigned to carbons CH_3_-19 and C-9 respectively; (iii) H-5α showed ^2^*J*_HC_ correlation with signals at *δ*_C_ = 29.79, 36.18 and 176.11 ppm assigned to carbons C-4, C-10 and C-6, respectively (Figure 3a). These 2D HMBC observations confirm unequivocally the 7-oxalactone position for compound **10**.

A similar analysis was performed to determine the structure of 6-oxolactone **13**. Thus, in the ^1^H-NMR spectrum a signal at *δ*_H_ = 4.46 ppm (1H, dd, *J* = 11.2 and 5.4 Hz) was assigned to H-5α, and correlated by 2D ^1^H-^13^C HSQC with the signal at *δ*_C_ = 79.53 ppm (CH with impair multiplicity from DEPT-135 spectrum). Additionally, H-5α showed ^2^*J*_HC_ correlation with signal at *δ*_C_ = 32.86 ppm that was assigned to the carbon C-4 and ^3^*J*_HC_ correlation with signals at *δ*_C_ = 11.48, 57.91 and 174.73 ppm, that were assigned to the carbons CH_3_-19, C-9 and C-6 (C=O, of lactone function, Table 1) respectively. While the signal of H-7 (2H, m) at *δ*_H_ = 2.51–2.39 ppm showed ^2^*J*_HC_ correlation with signals appearing at *δ*_C_ = 34.70 and 174.73 ppm, which were assigned to carbons C-8 and C-6 respectively (Figure 3b).

Saponification reaction of compounds **10** and **13** with K_2_CO_3_/MeOH in reflux and subsequent acidification with aqueous 5% HCl produced carboxylic acids **12** and **15** with 83% and 75% yields, respectively (Scheme 1). These compounds were previously reported, but only partial NMR spectroscopy data was provided [31]. Thus, a deeper NMR analysis for **12** and **15** is provided in Figure S1 of Supplementary Materials.

### 2.2. Bioactivity in the Rice Lamina Inclination Assay of Brassinosteroid Analogs

**Figure 4:**
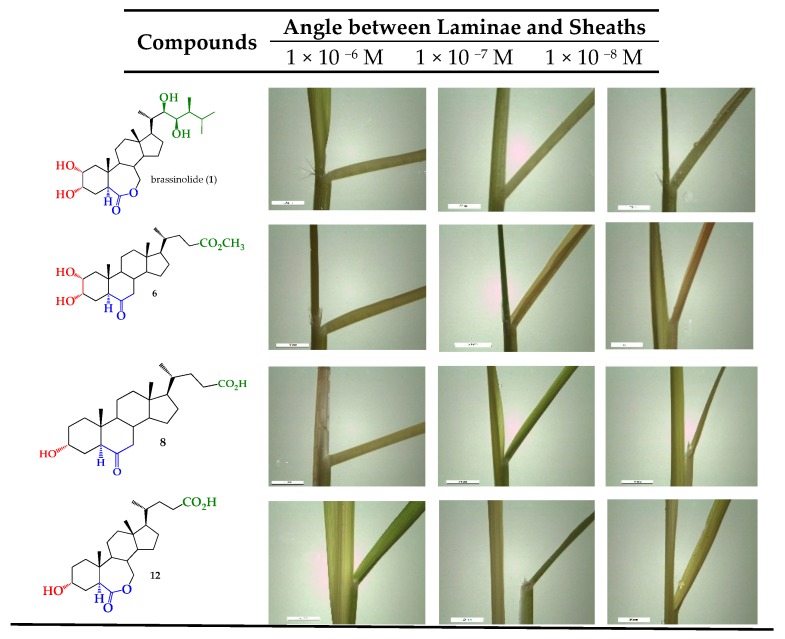
Rice-lamina assay using the second leaf lamina joints of excised leaf segments treated with brassinolide (**1**) and more active BR analogs at 1 × 10^−6^, 1 × 10^−7^ and 1 × 10^−8^ M. Scale bars = 2 cm.

A number of bioassays, such as first bean internode, root growth, and rice lamina inclination, has been developed to evaluate brassinolide activity [33,47,48,49,50,51,52]. In this work, plant growth-promoting activity of BR analogs was evaluated by the rice lamina inclination bioassay, because of its specificity and high sensitivity for brassinolide and related compounds [33,47,48,49], using a local rice (ZAFIRO) cultivar. The results are presented in Figure 4 and listed in Table 2.

The angles given in the table correspond to the difference between the induced angle produced by treatment with each compound and that found for the negative control. Values are means of angles measured for four segments per treatment considering two assays.

The values of Table 2 indicate that the effect is dose-dependent, i.e., for compounds **5**, **6** and **8** the activity increases steadily with increasing concentration, whereas compounds **12** and **15** exhibit an initial twofold increase in activity, which reach a maximum value at 1 × 10^−6^ M. Finally, the effect of brassinolide seems to level off above 1 × 10^−8^ M and then increases in a factor of two at 1 × 10^−6^ M.

The concentration effect on growth-promoting activity of BRs has been studied by several authors [53,54,55,56]. Wada et al. found a linear correlation between the bending of leaf segments and concentrations of brassinolide and castasterone in the range 1 × 10^−10^–1 × 10^−8^ M [53]. The effect leveled off at concentrations above 1 × 10^−8^ M. It has also been shown that application of exogenous BRs induces an inhibitory effect on root-growth in Arabidopsis when a threshold concentration is reached [54,55]. Similar results have been found in rice, where at relatively higher concentration BRs inhibit both root and seedling growth [57]. The effect of increasing concentrations of brassinolide on rice seedlings growth has also been evaluated, and the inhibitory effect has been attributed to inactivation of gibberellins, another growth hormone [58].

The opposite effects on growth of different tissues in the same plant, reported for high concentrations of exogenous BRs, has been explained in terms of differences in the concentration of endogenous BRs. Application of exogenous BRs induces growth inhibition in those tissues where the endogenous BR concentration is near to the threshold concentration, but promotes growth in those containing the lowest endogenous BR concentration [55].

As our results indicate that the concentration effect on growth activity depends on the structure of BR analogs, the activities measured at the lowest tested concentration (see Table 2) will be used to analyze the relation between activity and chemical structures of assayed compounds.

Brassinolide is commonly used as positive control to evaluate the activity of BR analogs, and therefore the effect of **1** on bending angle in the lamina assay has been taken as a reference. The results collected in Table 2 indicate that, at the lowest assayed concentration, compounds **12** and **15** are the most active BR analogs. The lowest activities are shown by deoxybrassinosteroids **5**, **6** and **8**, in which the oxa group is not present in ring B. These results are in line with other works where it has been suggested that the 2α,3α-dihydroxy-7-oxa-6-ketone moiety existing in brassinolide is a structural requisite for a compound manifesting the plant growth-promoting activity [33]. On the other hand, compounds **5** and **8** are synthetic analogs of typhasterol, which is considered to be a biosynthetic intermediate to castasterone and brassinolide [59], and whose activity in the rice-lamina inclination bioassay is only one tenth of that shown by brassinolide [60]. A comparison of activities of compounds **5** and **8** indicates that a minor change in the structure of the side alkyl chain (COOCH_3_ to COOH) induces a large increase in activity. On the other hand, a comparison of activities exhibited by analogs **12** and **15** shows that the 7-oxalactone is slightly more active than the 6-oxalactone. Similar results have been reported for other synthetic analogs of typhasterol, but the activity changes were 40–50 times larger for the 7-oxalactone [61]. In other words, a change in the spatial distribution of the oxa-lactone moiety in ring B produces a slight change in activity for these compounds.

Interestingly, at the higher assayed concentration deoxybrassinosteroids **6** and **8** become twofold more active than compounds **12** and **15**. However, as discussed above, these results are more difficult to explain because at higher concentrations there are other factors affecting the growth-promoting activity.

**Figure 5:**
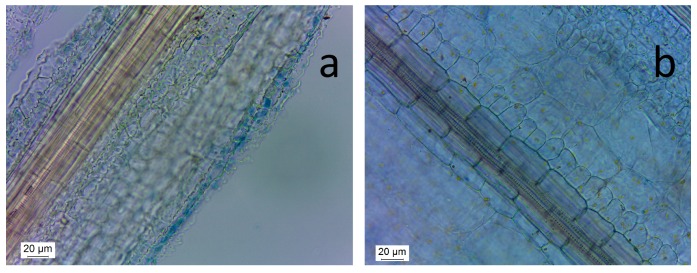
Changes induced by analog **8** in mesophyll cell length in second lamina of rice plants. Longitudinal sections of adaxial sides of the second leaf lamina joint: (**a**) negative control; (**b**) in the presence of analog **8**. Optic Microscope 40×, Scale of bars = 20 µm.

In order to determine the effect of BRs on cell elongation we have evaluated the effect of compound **8** on cell elongation by microscopy analysis. Frotis of vegetal tissue of second leaf lamina joints was performed, and the inner mesophyll cells were observed through an optical microscope 40×. The shape and size of cells showed that those cells submitted to treatment with compound **8** were stretched longitudinally as compared to the negative control (Figure 5a,b). These results indicate that growth-promoting effect of analog **8** is mainly due to changes of cell length of the adaxial sides, instead of an increase on cell number.

## 3. Materials and Methods

### 3.1. General

Chemicals were obtained from Merck (Darmstadt, Germany) or Aldrich (St. Louis, MO, USA) and were used without further purification. A detailed description of conditions used to register Fourier transform infrared (FT-IR) spectra, ^1^H, ^13^C, ^13^C DEPT-135, 2D HSQC and 2D HMBC spectra has been given elsewhere [26]. IR spectra were recorded as KBr disks in a FT-IR Nicolet 6700 spectrometer (Thermo Scientific, San Jose, CA, USA) and frequencies are reported in cm^−1^. High-resolution mass spectra (HRMS-ESI) were recorded in a Thermo Fisher Scientific Exactive Plus mass spectrometer. The analysis for the reaction products was performed with the following relevant parameters: heater temperature, 50 °C; sheath gas flow, 5 (arbitrary unit); sweep gas flow rate, 0 (arbitrary unit) and spray voltage, 3.0 kV at negative mode. The accurate mass measurements were performed at a resolving power: 140,000 FWHM at range *m*/*z* 300–500. Elemental analyses were obtained on a Carlo Erba Fisons EA-1108 Elemental Analyzer (Fisons Instruments/Carlo Erba Instruments, Milano, Italy). Silica gel (200–300 mesh, Merck, Darmstadt, Germany) was used for Column Chromatography (C.C.) and silica gel plates HF-254 for thin layer chromatography (TLC). TLC spots were detected by heating after spraying with 25% H_2_SO_4_ in H_2_O. Melting points were measured on a Stuart-Scientific SMP3 apparatus (Stone, Staffordshire ST15 OSA, UK) and are uncorrected.

### 3.2. Synthesis

#### 3.2.1. Methyl 2-en-6-oxo-5α-cholan-24-oate (**9**)

To a solution of compound **5** (1.0 g, 0.207 mmol) in 40 mL of CH_2_Cl_2_, 0.5 mL of pyridine and DMAP 10 mg (0.082 mmol) were added. Then the mixture was cooled to 0 °C in an ice-water bath. Later 1.2 mL (7.14 mmol) of trifluoromethanesulfonic anhydride (triflic anhydride) were added. The reaction mixture was kept under slow stirring at 0 °C for 24 h. The end of reaction was verified by TLC, the mixture was then concentrated in a rotary evaporator to a volume of approximately 10 mL. Then AcOEt (30 mL) was added and the organic layer was washed with NaHCO_3_ saturated solution (2 × 20 mL) and water (2 × 20 mL), dried over Na_2_SO_4_, and filtered. The solvent was evaporated in a rota-vapor and the crude was re-dissolved in CH_2_Cl_2_ (5 mL) and chromatographed on silica gel with EtOAc/hexane mixtures of increasing polarity (0.2:9.8 → 3.0:7.0) Compound **9** 0.793 g, 83% yield. The ^1^H, ^13^C-NMR and IR spectroscopic data and physical properties of alkene 9 were consistent with those reported [26,28,32].

#### 3.2.2. Methyl 2α, 3α-dihydroxy-6-oxo-5α-cholan-24-oate (**6**)

Compound **6** was synthesized from **9** according to the methodology previously described by us [26]. From 700 mg (1.81 mmol) of compound **9**, 517 mg (68% yield) were obtained. The ^1^H, ^13^C**-**NMR and IR spectroscopic data and physical properties of compound **6** were consistent with those reported [26,28,32]. Elemental analysis: found C, 71.45%; H, 9.67%; C_25_H_40_O_5_ requires C, 71.39%; H, 9.59%.

#### 3.2.3. Methyl 3α-acetoxy-6-oxo-5α-cholan-24-oate (**7**)

Compound **7** was synthesized from **5** by standard acetylation according to the methodology previously described by us [26]. From 3.5 g (8.65 mmol) of compound **7**, 3.78 g (98% yield) were obtained. The ^1^H, ^13^C**-**NMR and IR spectroscopic data and physical properties of compound **7** were consistent with those reported [26]. Elemental analysis: found C, 72.68%; H, 9.55%; C_27_H_42_O_5_ requires C, 72.61%; H, 9.48%.

#### 3.2.4. Methyl 3α-acetoxy-6-oxo-7-oxa-5α-cholan-24-oate (**10**) and Methyl 3α-acetoxy-6-oxa-7-oxo-5α-cholan-24-oate (**13**)

Preparation of oxidant: 0.40 mL of H_2_O_2_ (30%), (26.5 mmol) was slowly dripped into a solution of (CF_3_SO_2_)_2_O, (16.9 mL, 119.9 mmol) at 0 °C, diluted with CHCl_3_ (3 mL) and stirred for 30 min.

The oxidant mixture was slowly added over the solution of compound 7 (0.8 g, 1.79 mmol in 10 mL of CHCl_3_) at 0 °C and slowly stirred in N_2_ atmosphere for 24 h. The end of reaction was verified by TLC, the mixture was filtered, then concentrated in a rotary evaporator to a volume of approximately 10 mL. Then AcOEt (40 mL) was added and the organic layer was washed with saturated NaHCO_3_ solution (2 × 20 mL), water (2 × 15 mL), then dried over Na_2_SO_4_, and filtered. The solvent was evaporated and the crude was re-dissolved in CH_2_Cl_2_ (5 mL) and chromatographed on silica gel with EtOAc/hexane mixtures of increasing polarity (0.2:50.0 → 23.8:26.2) Three fractions were obtained. Fraction I: 0.35 g (44% yield) compound 10. Fraction II: 0.27 g of mixture compounds 10 and 13. Fraction III: 0.066 g (8% yield) compound 13.

Compound **10**. Colorless solid (m.p. = 131 ± 2 °C, Hexane/Et_2_O) IR (cm^−1^): 2949 (C–H); 2867 (C–H); 2849 (C–H); 1738 (C=O); 1366 (CH_3_); 1240 (C–O); 1189 (C–O); 1080 (C–O). ^1^H-NMR (CDCl_3_): 5.09 (1H, m, H-3); 4.10–4.07 (2H, m, H-7); 3.66 (s, 3H, CH_3_O); 3.02 (1H, dd, *J* = 12.1 and 4.0 Hz, H-5); 2.35 (1H, m, H-23); 2.26–2.13 (2H, m, H-23 and H-4); 2.06 (3H, s, CH_3_CO); 0.92 (3H, d, *J* = 6.5 Hz, H-21); 0.89 (3H, s, H-19); 0.69 (3H, s, H-18). ^13^C-NMR: See Table 1. Elemental analysis: found C, 70.16%; H, 9.23%; C_27_H_42_O_6_ requires C, 70.10%; H, 9.15%.

Compound **13**. Colorless solid (m.p. = 125 ± 3 °C, Hexane/Et_2_O) IR (cm^−1^): 2978 (C–H); 2955 (C–H); 2863 (C–H); 1738 (C=O); 1720 (C=O); 1365 (CH_3_); 1254 (C–O); 1038 (C–O). ^1^H-NMR (CDCl_3_): 5.06 (1H, m, H-3); 4.46 (1H, dd, *J* = 11.2 and 5.4 Hz, H-5); 3.66 (3H, s, CH_3_O); 2.51–2.39 (2H, m, H-7); 2.35–2.27 (1H, m, H-23); 2.22–2.18 (1H, m, H-23); 2.03 (3H, s, CH_3_CO); 0.870 (3H, d, *J* = 4.8 Hz, H-21); 0.865 (3H, s, H-19); 0.650 (3H, s, H-18). ^13^C-NMR: See Table 1. HRMS-ESI (negative mode): Calculated 405.2719 found 405.2762. Elemental analysis: found C, 70.18%; H, 9.25%; C_27_H_42_O_6_ requires C, 70.10%; H, 9.15%.

#### 3.2.5. Acid-3α-hydroxy-6-oxo-7-oxa-5α-cholan-24-oic (**12**)

To a solution of compound **10** (100 mg, 0.22 mmol) in 20 mL of CH_3_OH, 10 mL of 15% aqueous solution of K_2_CO_3_ were added. The reaction mixture was maintained with constant stirring and reflux for 1 h. The end of reaction was verified by TLC, then the mixture was concentrated in a rotary evaporator and evaporated to dryness. The crude was acidified with 15 mL of aqueous 5% HCl (until pH = 2–3). The solid obtained was filtered and washed with water (10–15 mL) to neutral pH and then it was dried under a vacuum. 73 mg (83% yield) of compound **12** were obtained. Compound **12**: colorless solid (m.p. = 177–180 °C, MeOH/Et_2_O; referential: 177–181 °C [24]) IR (cm^−1^): 3480–2540 (O–H); 2926 (C–H); 2869 (C–H); 2853 (C–H); 1725 (C=O); 1704 (C=O); 1380 (CH_3_); 1189 (C–O); 1128 (C–O); 1074 (C–O). ^1^H-NMR (CD_3_OH): 4.21 (1H, dd, *J* = 12.5 and 9.8 Hz, H-7a), 4.08–4.03 (2H, m, H-7b and H-3); 3.24 (1H, dd, *J* = 12.5 and 4.2 Hz, H-5α); 2.33–2.29 (1H, m, H-23); 2.23–2.07 (1H, m, H-23); 0.95 (3H, d, *J* = 6.4 Hz, H-21); 0.86 (3H, s, H-19); 0.740 (3H, s, H-18). ^13^C-NMR: See Table 1. HRMS-ESI (negative mode): Calculated 405.2719 found 405.2650. Elemental analysis: found C, 70.98%; H, 9.50%; C_24_H_38_O_5_ requires C, 70.90%; H, 9.42%.

#### 3.2.6. Acid-3α-hydroxy-6-oxa-7-oxo-5α-cholan-24-oic (**15**)

Compound **15** was obtained from **13** by the same method described above. To a solution of compound **13** (50 mg, 0.11 mmol) in 15 mL of CH_3_OH, 5 mL of 15% aqueous solution of K_2_CO_3_ were added. Then 33 mg (75% yield) of compound **15** were obtained. Compound **15**: colorless solid (m.p. = 275–279 °C, MeOH/Et_2_O; referential: 276–278 °C [31]) IR (cm^−1^): 3457–2538 (O–H); 2956 (C–H); 2943 (C–H); 2870 (C–H); 2852 (C–H); 1730 (C=O); 1680 (C=O); 1380 (CH_3_); 1250 (C–O); 1199 (C–O); 1140 (C–O); 1074 (C–O). ^1^H-NMR: (CD_3_OD) 4.68 (1H, dd, *J* = 11.0 and 5.2 Hz, H-5α); 4.09 (1H, b.s, H-3); 2.61 (1H, dd, *J* = 12.6 and 12.0 Hz, H-7a); 2.40 (1H, d, *J* = 12.0 Hz, H-7b); 2.30 (1H, m, H-23); 2.19 (1H, m, H-23); 0.940 (3H, d, *J* = 6.4 Hz, H-21); 0.880 (3H, s, H-19); 0.730 (3H, s, H-18). ^13^C-NMR: See Table 1. Elemental analysis: found C, 70.96%; H, 9.49%; C_24_H_38_O_5_ requires C, 70.90%; H, 9.42%.

### 3.3. Biological Activity: A Rice Lamina Inclination Assay

The lamina joint bending assay using excised leaf segments was performed as previously described [33] with minor modifications. After soaking for 24 h in distilled water, seeds of a rice (*Oryza sativa*) cultivar Zafiro (obtained from the National Agricultural Research Institute (INIA)) were grown in pots (10 cm length × 15 cm diameter) filled with substrate + vermiculite + perlite (in the ratio 2:1:1) inside a greenhouse maintained at a 16 h day (22 °C)/8 h night (20 °C) cycle, with 50%–60% of relative humidity (RH). Uniform seedlings were selected and leaf segments of approximately 8 cm, consisting of the second leaf lamina and the second lamina joint and sheath, were excised. For each treatment, four of these segments were incubated in 60 mL of sterile distilled water for 24 h inside a petri dish containing a finite amount (1 × 10^−8^, 1 × 10^−7^ and 1 × 10^−6^ M) of the test sample (BR analogs: **5**, **6**, **8**, **12** and **15**). Brassinolide, used as positive control, was purchased from Sigma Chemical Co., St. Louis, USA. The negative control contains only physiological saline. After incubating for 48 h at 25 °C in darkness, the magnitude of the angle between the leaf and sheath was measured. Images were taken using a Leica EZ4HD Stereo Microscope with camera software. This test was made in duplicate for each treatment.

Tissue sections from leaf segments treated with compound **8** were fixed, stained with Lactophenol Cotton Blue (BD, Franklin Lakes, NJ, USA), and observed by optical microscopy. Images were captured with a camera ICC50HD coupled to a DM500 microscope (Leica Microsystems, Wetzlar, Germany), and cell lengths were measured using Leica software ((Leica Microsystems, Wetzlar, Germany).

## 4. Conclusions

Therefore, active BR analogs synthesized and described in this work might be used as an alternative to manipulate plant height and offer the potential to improve production of crops. However, the optimal growth-promoting concentration must be determined.

## Figures and Tables

**Table 1:** *δ*^13^C-RMN (CDCl_3_, 100.6 MHz) for compounds **10**, **12**, **13** and **15**.

C	10	12 *	13	15 *
1	33.69	34.04	31.74	32.40
2	27.84	28.90	27.37	28.51
3	68.48	65.49	69.43	66.95
4	29.79	33.39	32.86	36.57
5	42.64	42.88	79.53	81.59
6	176.11	179.68	174.73	178.28
7	70.47	71.75	38.02	38.79
8	39.43	40.79	34.70	36.36
9	58.43	59.49	57.91	59.09
10	36.18	37.25	39.58	40.88
11	22.15	23.26	22.09	23.27
12	39.64	41.01	39.52	40.97
13	42.66	43.82	42.61	43.87
14	55.81	57.12	56.00	57.46
15	25.17	28.83	25.15	28.51
16	24.80	25.76	24.75	26.32
17	51.52	52.54	55.37	56.54
18	11.79	12.21	11.67	12.15
19	14.58	14.93	11.48	11.83
20	35.32	36.62	35.18	36.62
21	18.14	18.63	18.04	18.62
22	30.81	31.95	30.66	31.97
23	31.03	32.15	30.90	32.12
24	174.59	178.09	174.39	178.20
CH_3_O	51.46	-	51.39	-
CH_3_CO-	170.32	-	170.02	-
CH_3_CO-	21.38	-	21.19	-

* The ^13^C-NMR spectrum of compounds **12** and **15** was recorded in CD_3_OD solution.

**Table 2:** Effect of brassinolide and analogs **5**, **6**, **8**, **12** and **15**, on the lamina inclination of rice seedlings.

BRs	Angle Degrees between Laminae and Sheaths (°) (± Standard Error)		
	Concentration (M)		
Compounds	1 × 10^−6^	1 × 10^−7^	1 × 10^−8^
**5**	11 ± 2.5	1 ± 0.0	0 ± 0.0
**6**	47 ± 4.7	29 ± 4.5	0 ± 1.0
**8**	49 ± 2.0	29 ± 6.3	7 ± 2.4
**12**	26 ± 2.5	26 ± 2.5	15 ± 0.0
**15**	23 ± 6.0	18 ± 8.7	10 ± 0.0
Brassinolide (C+)	64 ± 4.8	30 ± 4.1	28 ± 2.9
Control (C−)	20 ± 0		

## Supplementary Materials

Supplementary materials can be found at www.mdpi.com/1422-0067/18/3/516/s1.

## Abbreviations

MDPI	Multidisciplinary digital Publishing Institute
DOAJ	Directory of open access journals
TLA	Three letter acronym
LD	Linear dichroism
BRs	Brassinosteroids
DCM	Dichloromethane
PCC	Pyridinium ChloroChromate
DMAP	4-Dimethylaminopyridine
DMF	Dimethylformamide
Tf_2_O	Trifluoromethanesulfonic anhydride
*m*-CPBA	3-Chloro or *metha*-chloroperoxybenzoic acid
DEPT-135	Distortionless Enhancement by Polarization Transfer with flip angle of 135°
HSQC	Heteronuclear Single Quantum Coherence
HMBC	Heteronuclear Multiple Bond Correlation
