# Supplementary material for: Synthesis of Five Known Brassinosteroid Analogs from Hyodeoxycholic Acid and Their Activities as Plant-Growth Regulators
Source: Int J Mol Sci. 2017 Mar 8;18(3):516. doi: 10.3390/ijms18030516 (PMC5372532; doi:10.3390/ijms18030516)
Supplement: Supplementary file 1 [file ijms-18-00516-s001.pdf]

Supplementary Materials: Synthesis of Five Known Brassinosteroid Analogs from Hyodeoxycholic Acid and Their Activities as Plant-Growth Regulators

María Isabel Duran, Cesar González, Alison Acosta, Andrés F. Olea, Katy Díaz and Luis Espinoza

FTIR and full NMR Spectra of Compounds **10**, **13**, **12** and **15**.

Methyl 3α-acetoxy-6-oxo-7-oxa-5α-cholan-24-oate (**10**)

FTIR

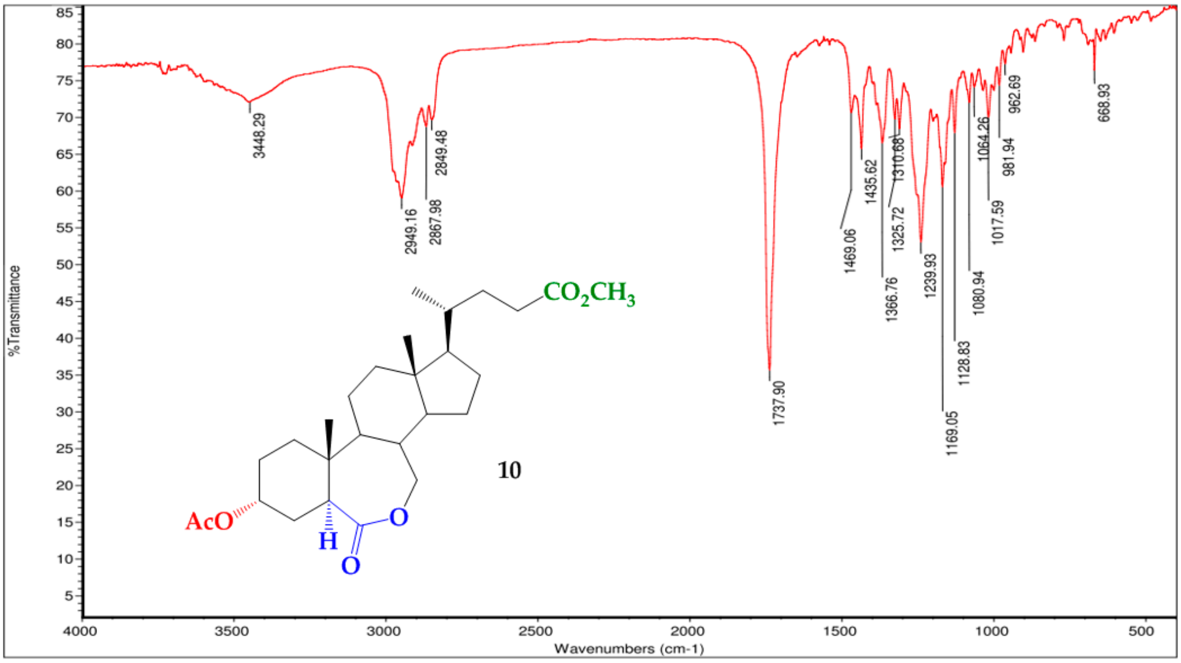

^{1}H-NMR

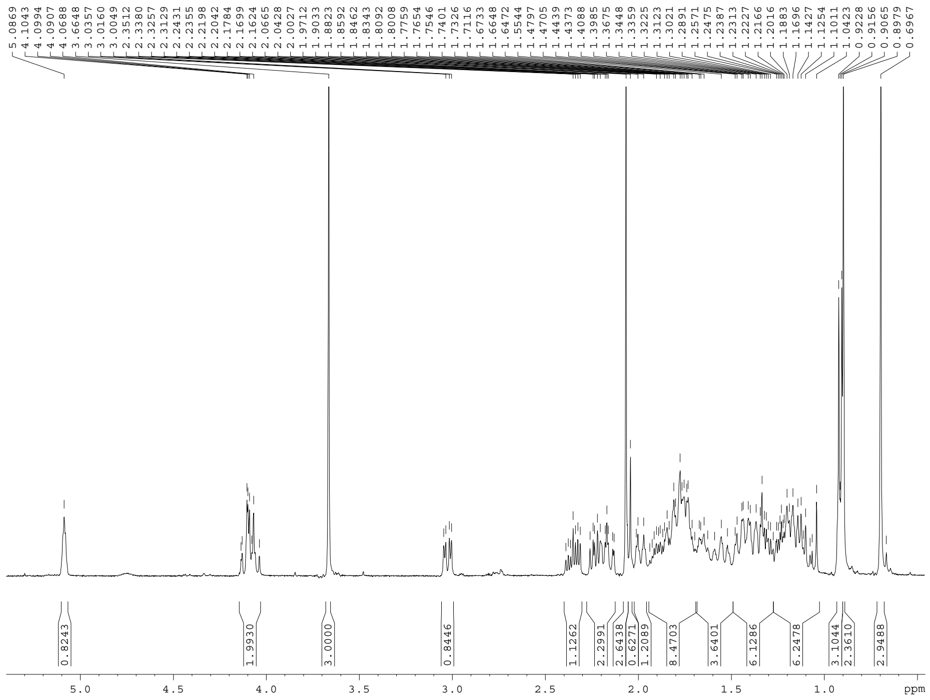

^{13}C-NMR

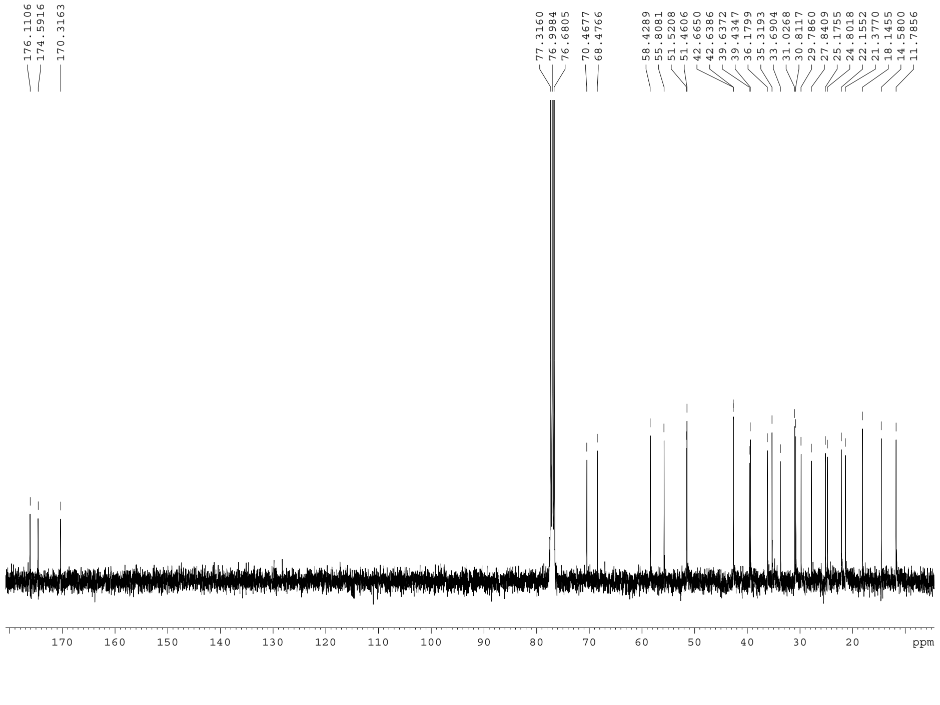

^{13}C DEPT-135 NMR

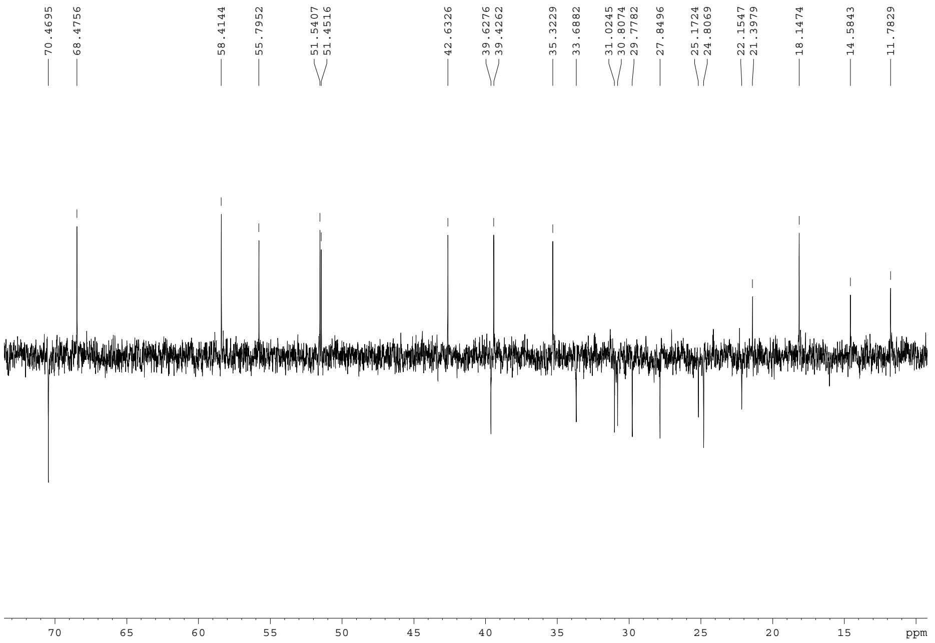

^{1}H-^{13}C 2D HSQC

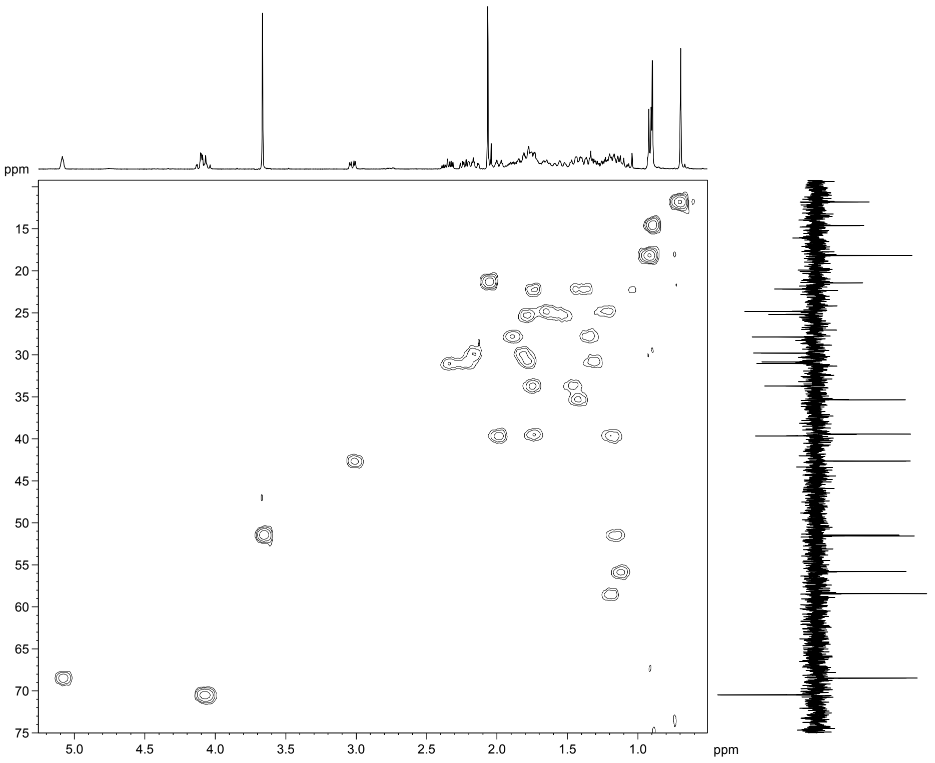

¹H-¹³C 2D HMBC

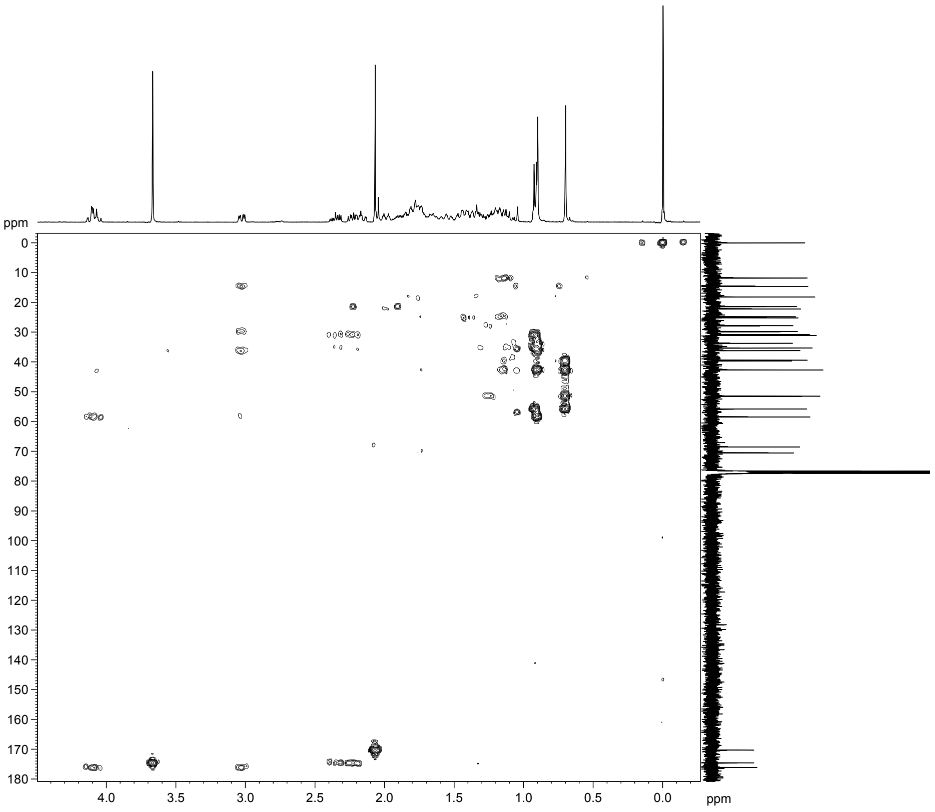

*Methyl 3α-acetoxy-6-oxa-7-oxo-5α-cholan-24-oate (**13**).*

FTIR

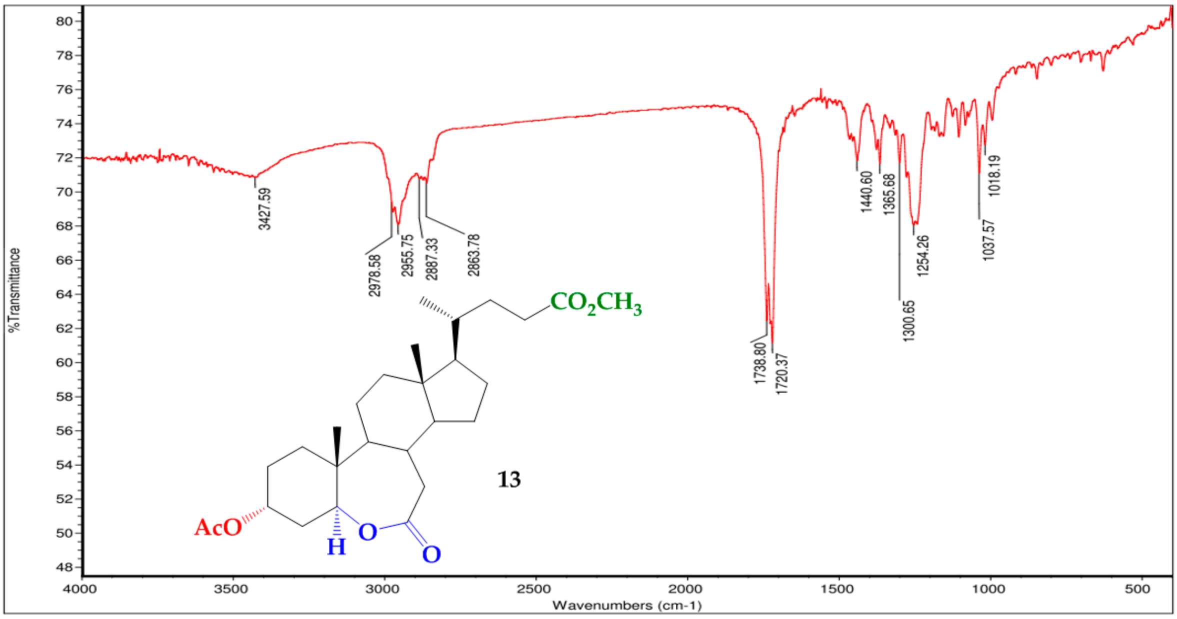

^{1}H-NMR

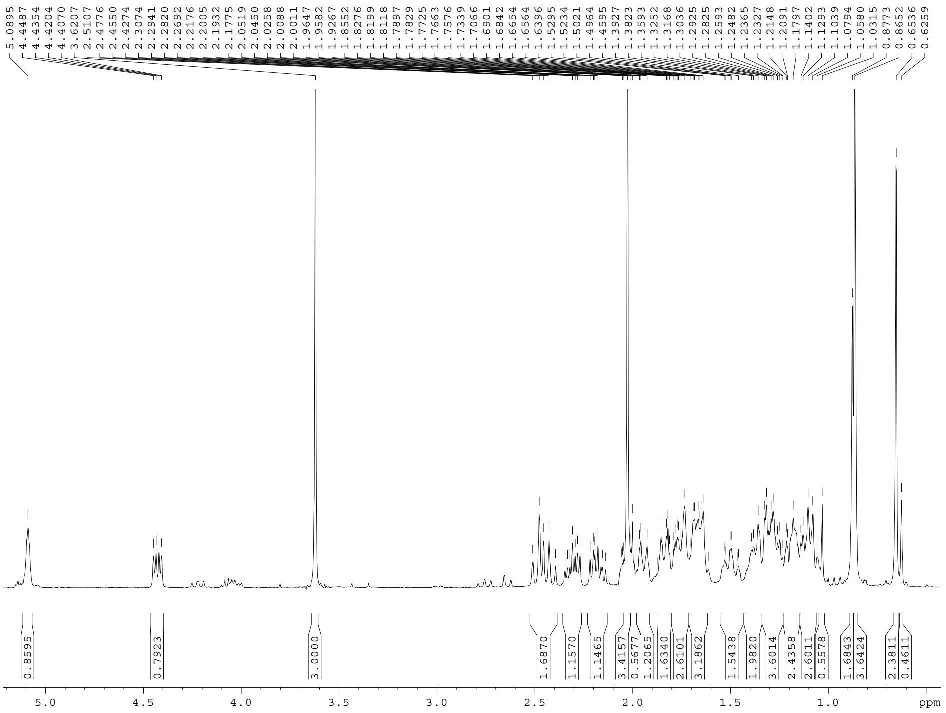

^{13}C-NMR

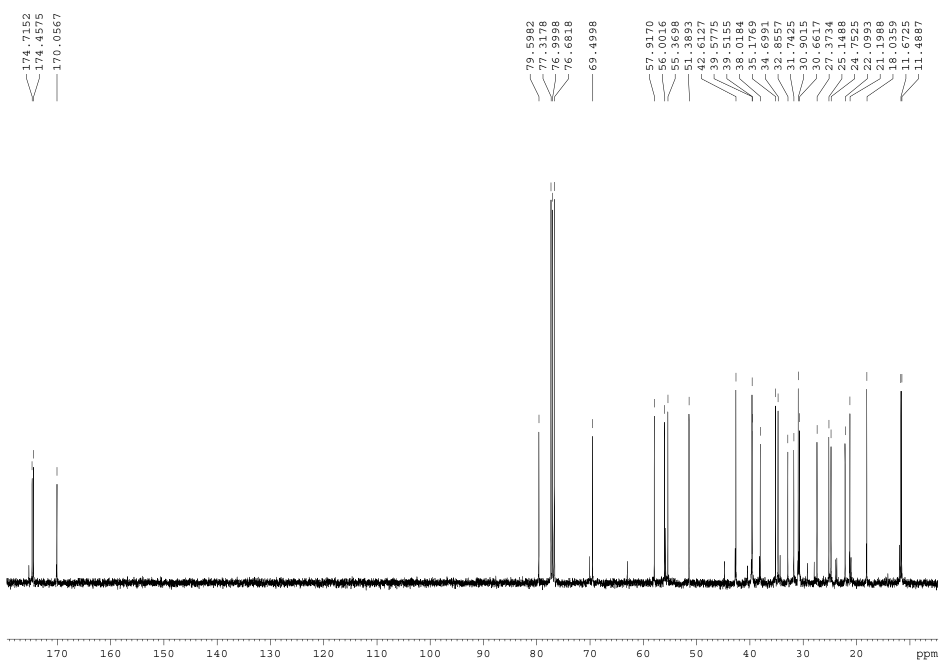

^{13}C DEPT-135 NMR

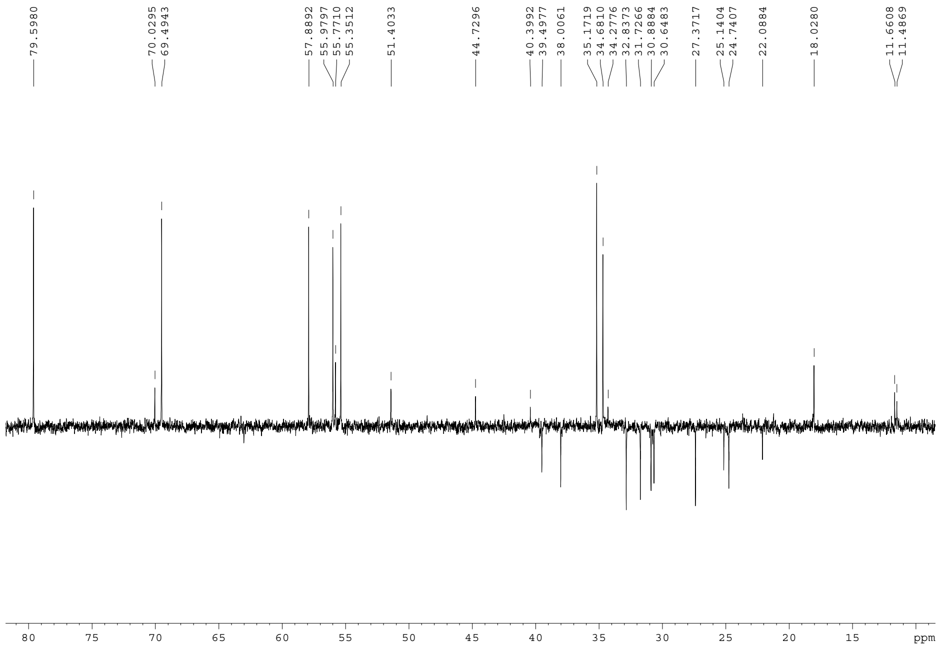

^{1}H-^{13}C 2D HSQC

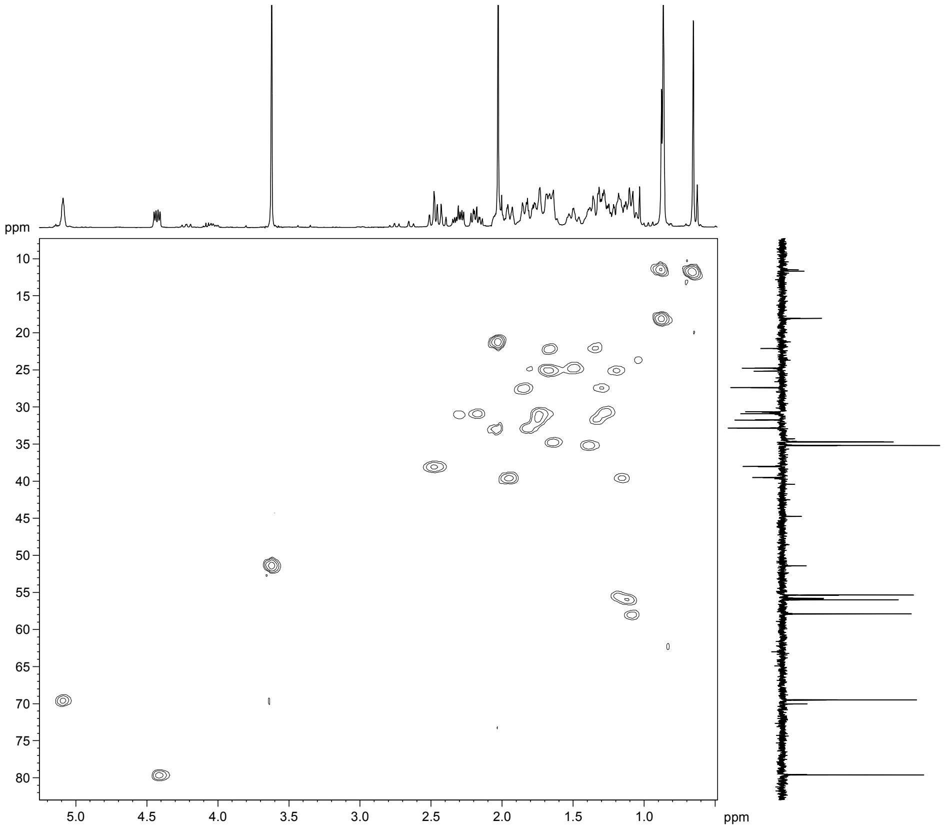

^{1}H-^{13}C 2D HMBC

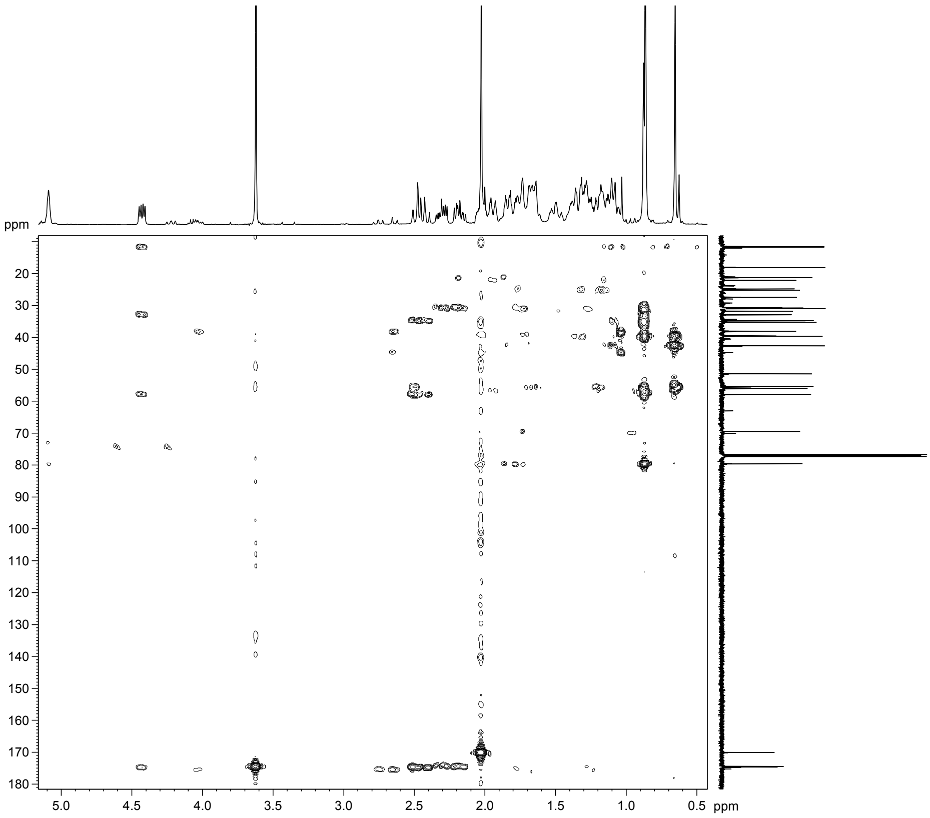

*Acid-3α-hydroxy-6-oxo-7-oxa-5α-cholan-24-oic (**12**)*

FTIR

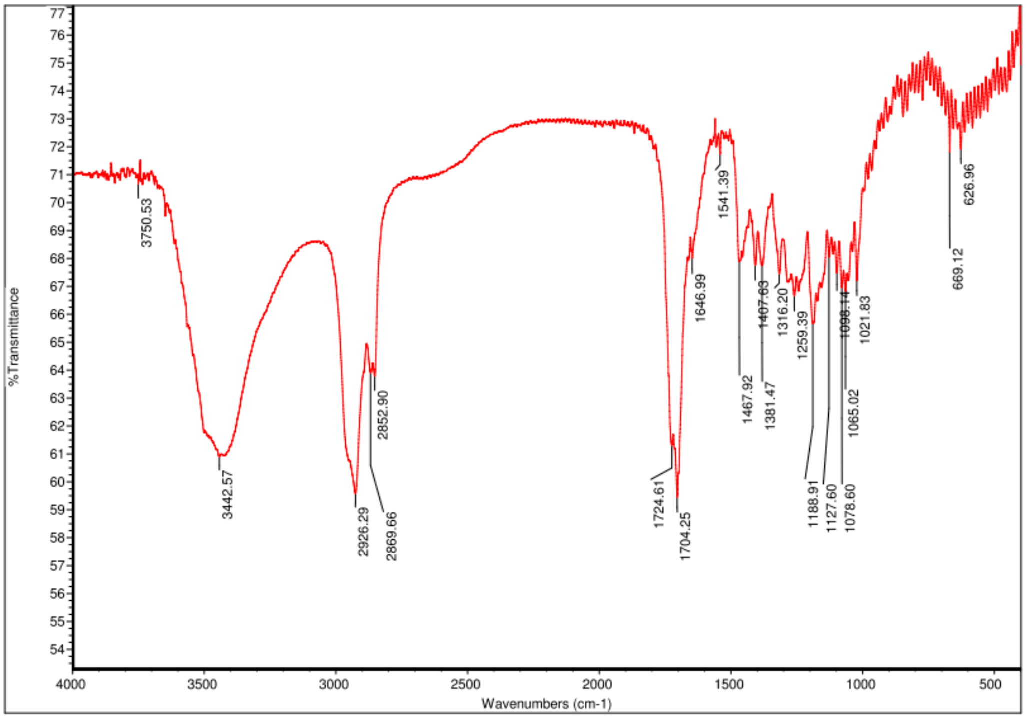

^{1}H-NMR

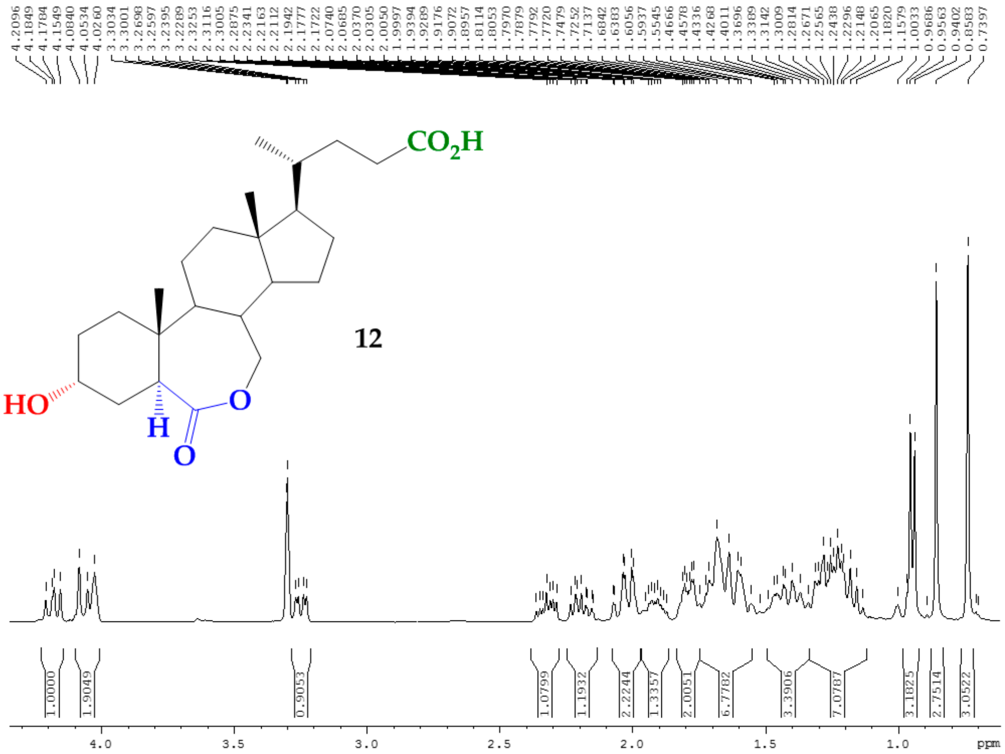

^{13}C-NMR

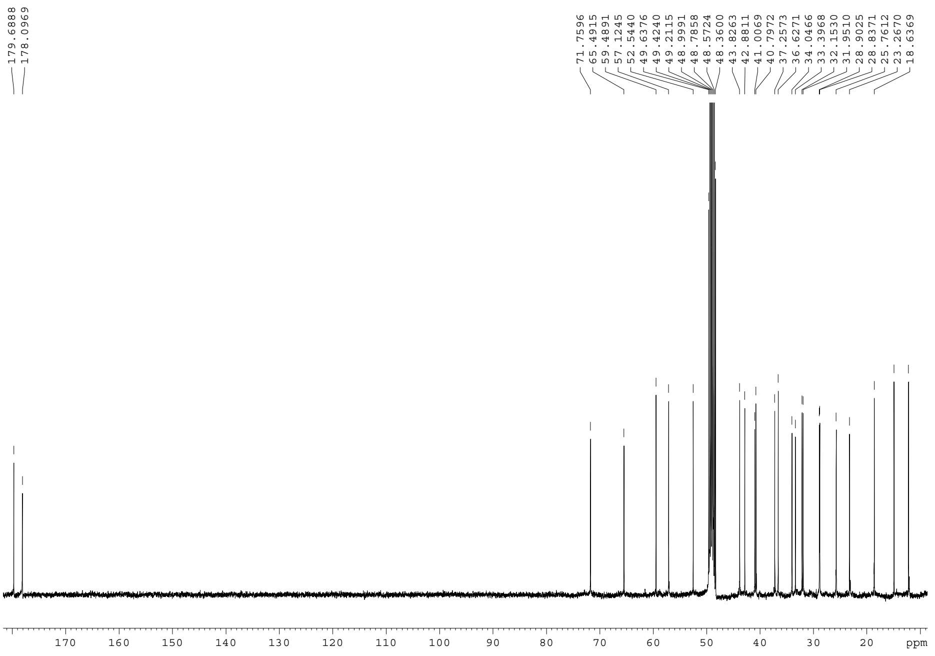

¹³C DEPT-135 NMR

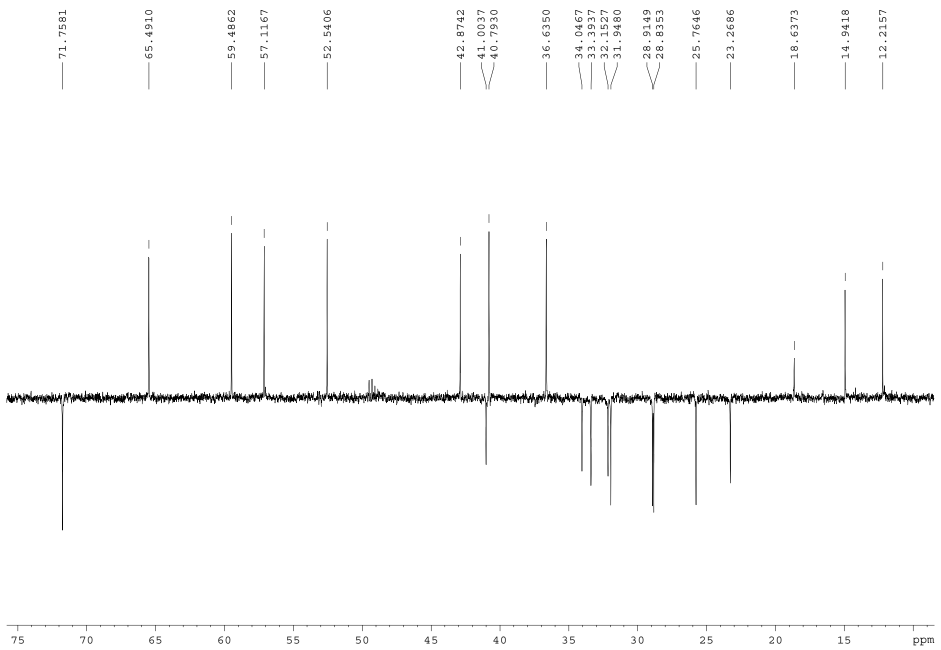

¹H-¹³C 2D HSQC

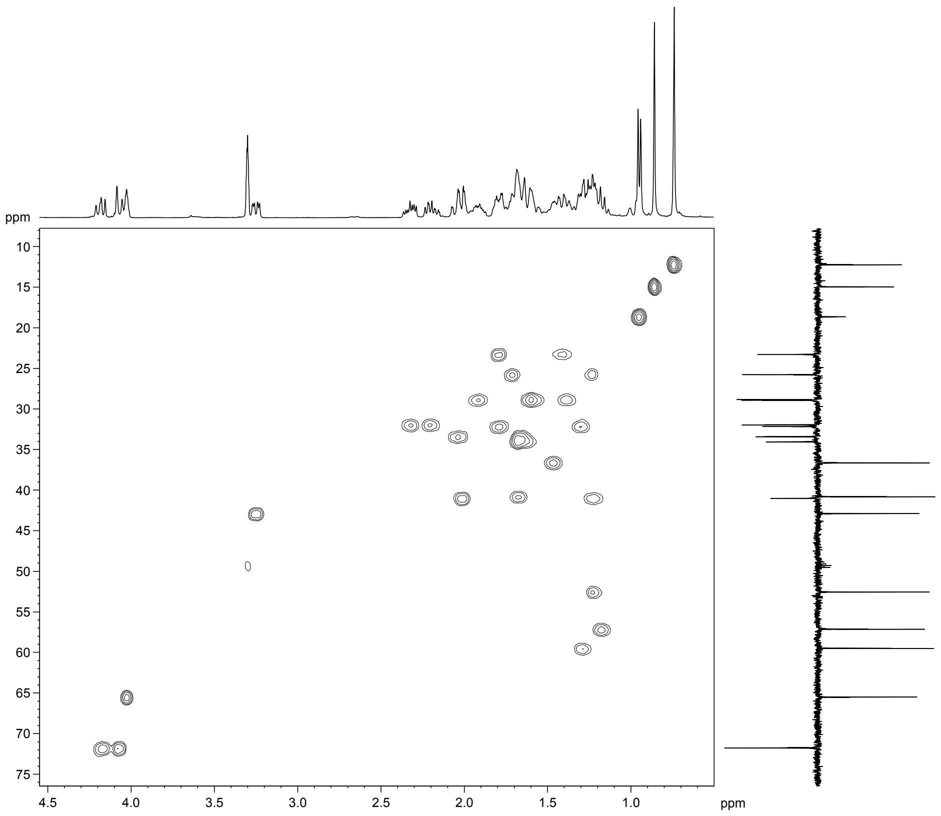

^{1}H-^{13}C 2D HMBC

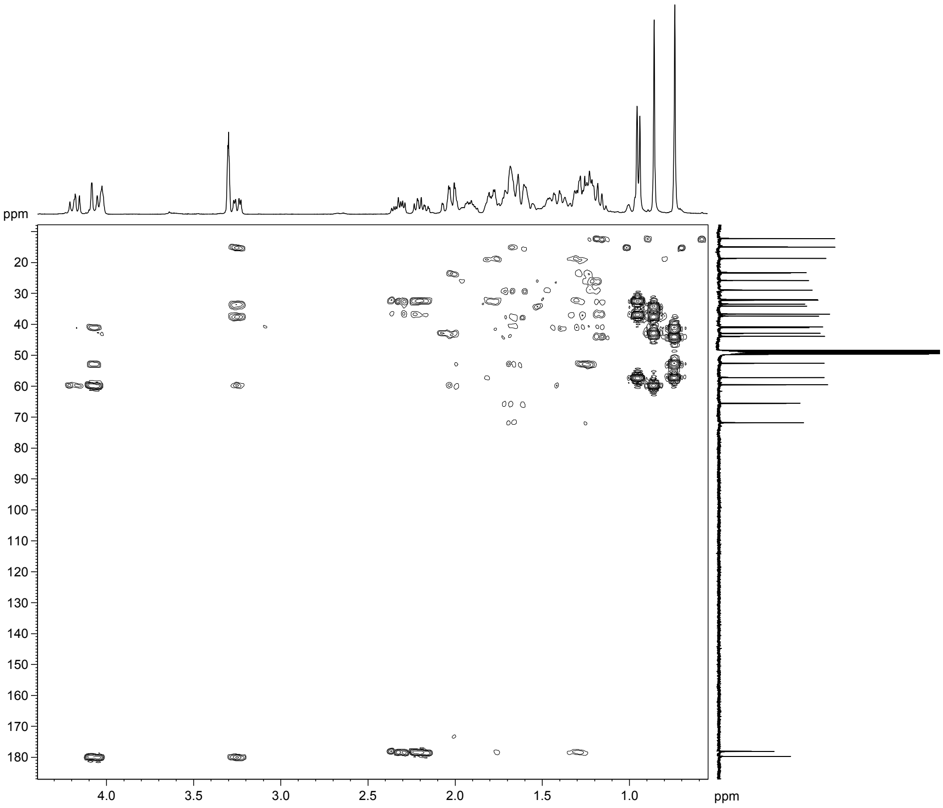

Acid-3α-hysroxy-6-oxa-7-oxo-5α-cholan-24-oic (**15**)

FTIR

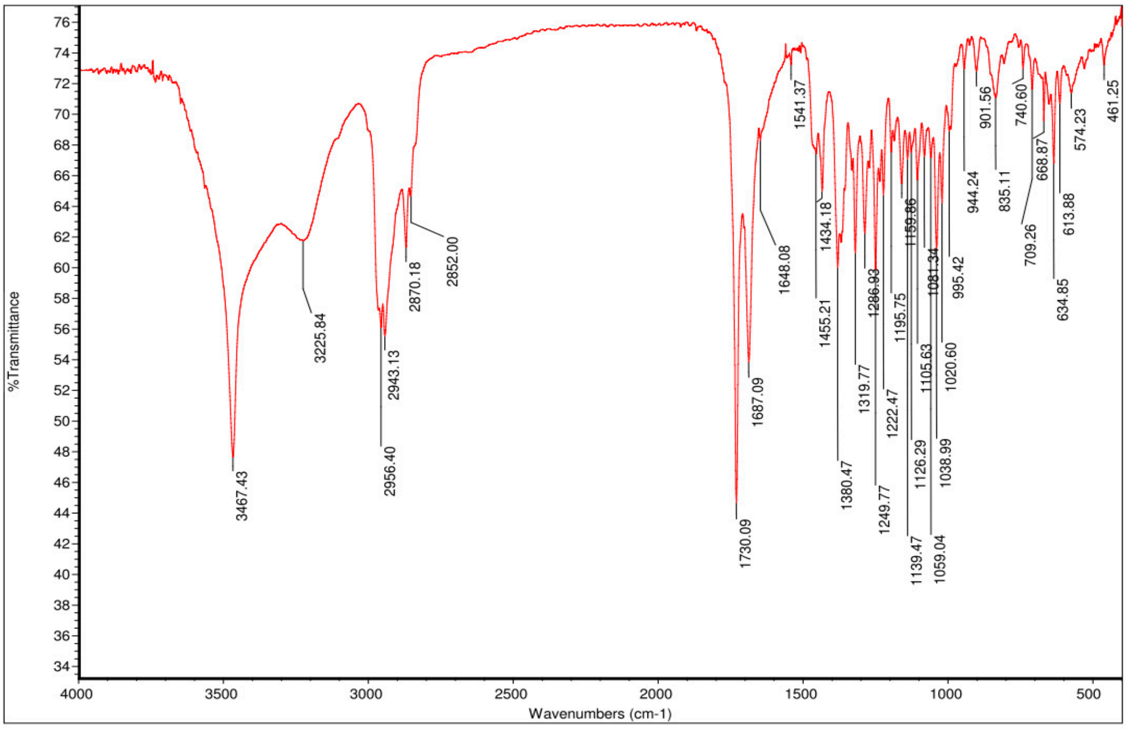

^{1}H-NMR

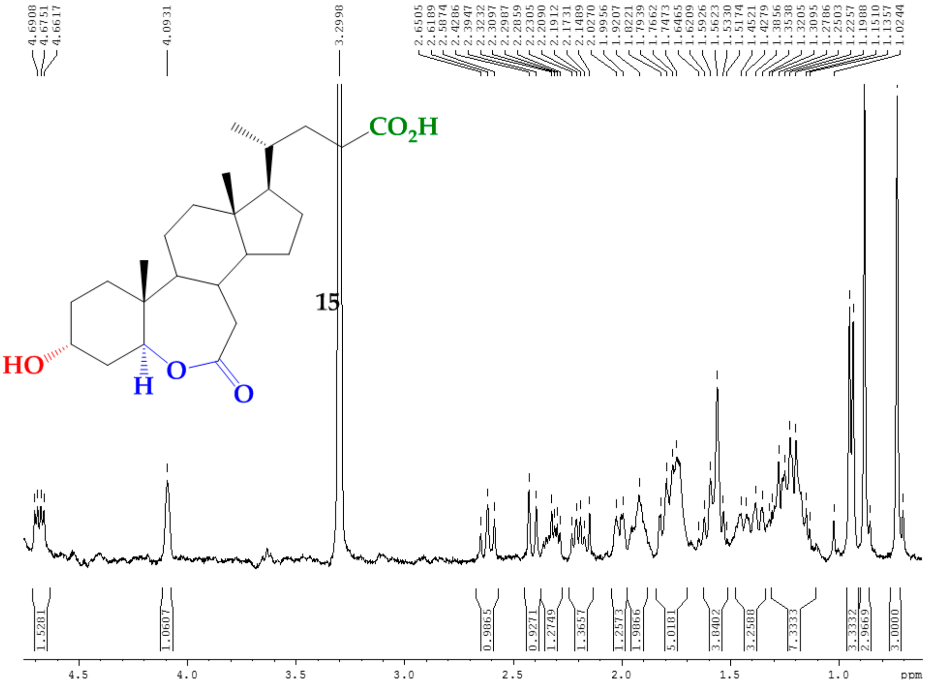

^{13}C-NMR

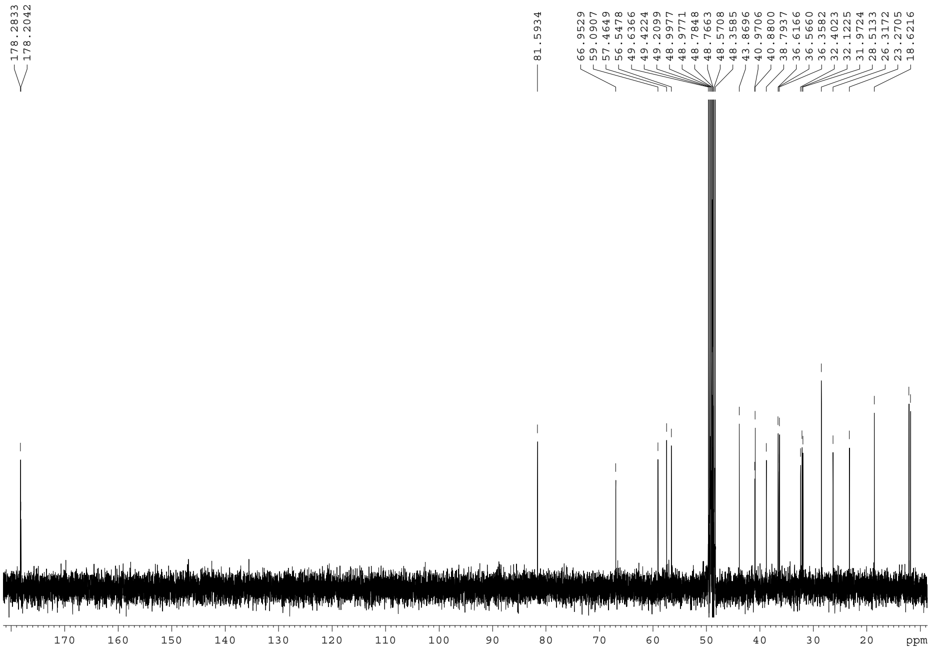

^{13}C DEPT-135 NMR

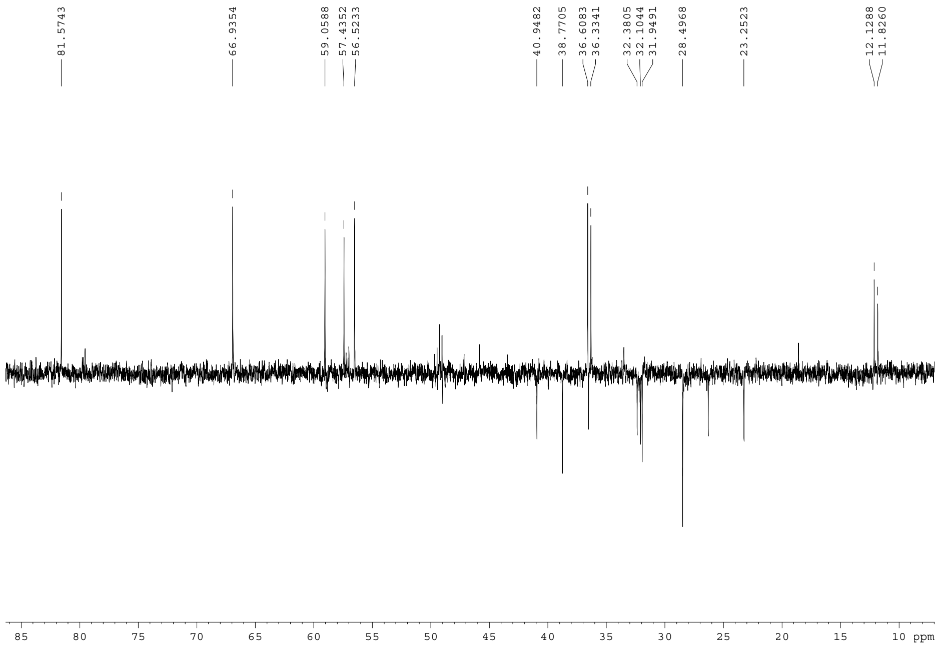

^{1}H-^{13}C 2D HSQC

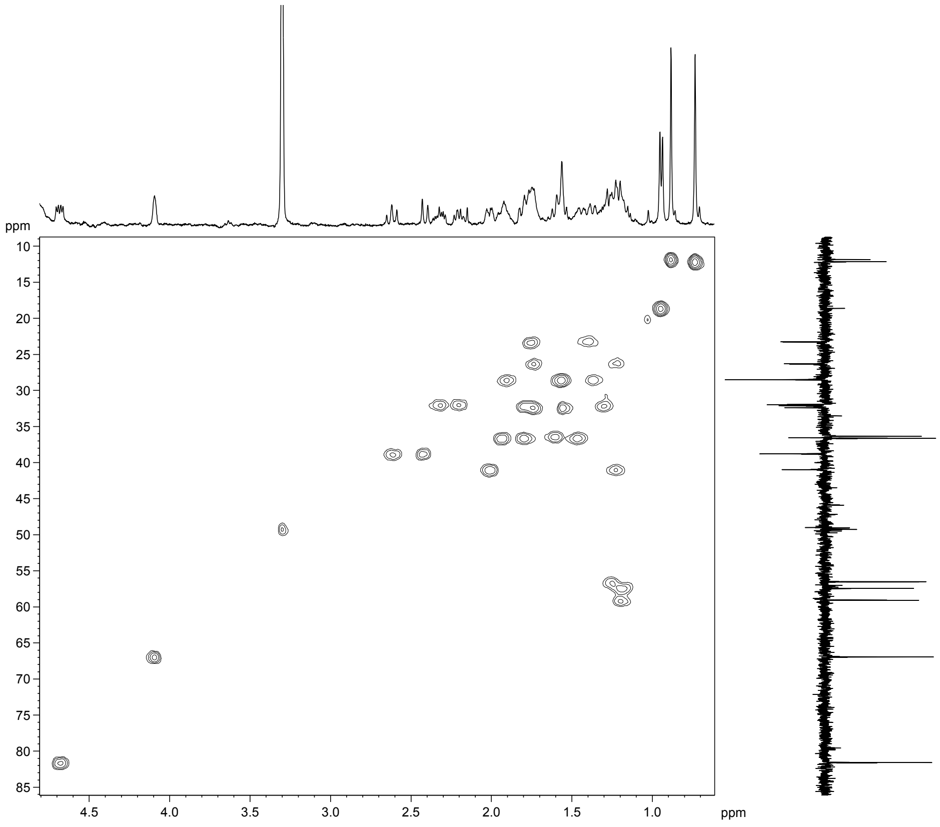

^{1}H-^{13}C 2D HMBC

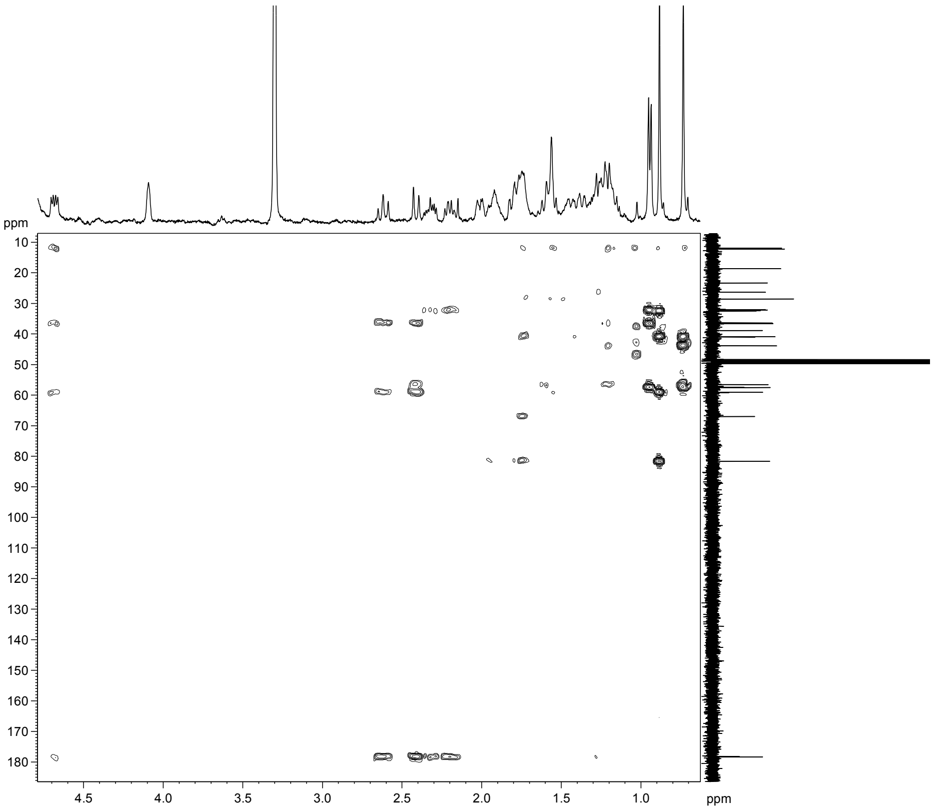

In the ^{1}H-NMR spectrum of compound **12** a signal was observed at δ_H = 4.21 ppm (1H, dd, *J* = 12.5 and 9.8 Hz), assigned to hydrogen H-7a, and correlated by 2D ^{1}H-^{13}C HSQC with the signal δ_C = 71.75 ppm (CH_2-7 from ^{13}C and DEPT-135 spectra). Signals at δ_H = 4.08-4.03 ppm (2H, m) were assigned to hydrogen atoms H-7b and H-3, correlated with the signals δ_C = 71.75 and 65.49 ppm (Table 1), respectively (by 2D HSQC and DEPT-135 spectra). Signals at δ_H = 2.33-2.29 ppm (1H, m) and δ_H = 2.23-2.07 ppm (1H, m) were assigned to hydrogen H-23 and correlated with the signal δ_C = 32.15 ppm by 2D HSQC, ^{13}C and DEPT-135 spectra. Signals at δ_H = 0.95 ppm (3H, d, *J* = 6.4 Hz), δ_H = 0.86 ppm (3H, s) and δ_H = 0.74 ppm (3H, s) were assigned to methyl groups CH_3-21, CH_3-19 and CH_3-18, respectively. Additionally, H-5α at δ_H = 3.24 ppm (1H, dd, *J* = 12.5 and 4.2 Hz) showed $^{2}J_{HC}$ 2D HMBC correlation with signal at δ_C = 33.39 ppm that was assigned to carbon C-4 and with signal at δ_C = 179.68 ppm, assigned to the carboxylic group of lactone function (C-6) (Figure S1a). H-5α also showed $^{3}J_{HC}$ correlation with signals at δ_C = 14.93, 34.04 and 59.49 ppm, which were assigned to carbons CH_3-19, C-1 and C-9, respectively. The signal of H-7a (δ_H = 4.21 ppm) shows a correlation at $^{3}J_{HC}$ with signal at δ_C = 59.49 ppm (C-9), whereas the signal of H-7b (δ_H = 4.08-4.03 ppm) showed $^{2}J_{HC}$ correlation with signal at δ_C = 40.79 ppm (C-8) and $^{3}J_{HC}$ with signals at δ_C = 52.54, 59.49 and 179.68 ppm (Table 1), which were assigned to carbons C-17, C-9 and C-6, respectively (Figure S1a).

A similar analysis was performed for structure assignment of compound **15**. Thus, hydrogen H-5α at δ_H = 4.68 ppm (1H, dd, *J* = 11.0 and 5.2 Hz) correlated by 2D HSQC with signal at δ_C = 81.59 ppm (C-5), and also showed $^{2}J_{HC}$ 2D HMBC correlation with signal at δ_C = 36.57 (C-4) and $^{3}J_{HC}$ with signals at δ_C = 11.83, 59.09 and 178.28 ppm (Table 1), which have been assigned to carbons C-19, C-9 and carboxylic group of lactone function (C-6) (Figure S1b). Signal of hydrogen H-7a at δ_H = 2.61 ppm (1H, dd, *J* = 12.6 and 12.0 Hz) showed $^{2}J_{HC}$ correlation with signals at δ_C = 36.36 ppm (C-8) and 178.28 ppm (C-6). The hydrogen H-7b at δ_H = 2.40 ppm (1H, d, *J* = 12.0 Hz) also showed $^{2}J_{HC}$ correlations with C-8 and C-6 and $^{3}J_{HC}$ with signals at δ_C = 56.54 ppm (C-17) and C-9 (Figure S1b).

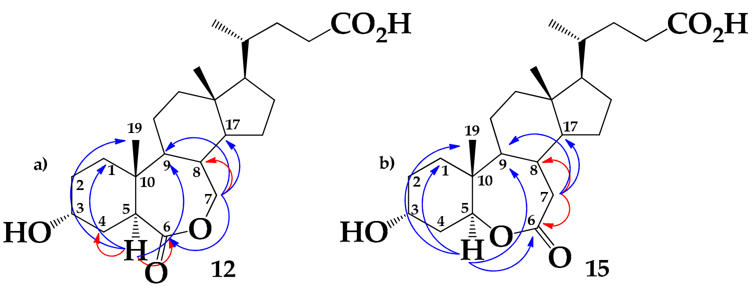

Figure S1. Major correlations observed for compounds **12** (a) and **15** (b); $^{2}J_{HC}$ (**red arrows**) and $^{3}J_{HC}$ (**blue arrows**) of hydrogens H-5α and H-7.
